# Glucagon-Like Peptide-2 and the Enteric Nervous System Are Components of Cell-Cell Communication Pathway Regulating Intestinal Na^+^/Glucose Co-transport

**DOI:** 10.3389/fnut.2018.00101

**Published:** 2018-10-26

**Authors:** Andrew W. Moran, Miran A. Al-Rammahi, Daniel J. Batchelor, David M. Bravo, Soraya P. Shirazi-Beechey

**Affiliations:** ^1^Department of Functional and Comparative Genomics, Institute of Integrative Biology, University of Liverpool, Liverpool, United Kingdom; ^2^Department of Medical Biotechnology, College of Biotechnology, University of Al-Qadisiyah, Al-Diwaniyah, Iraq; ^3^Pancosma SA, Geneva, Switzerland

**Keywords:** SGLT1, regulation, glucose, intestine, GLP-2

## Abstract

The Na^+^/glucose cotransporter 1, SGLT1 is the major route for transport of dietary glucose from the lumen of the intestine into absorptive enterocytes. Sensing of dietary sugars and artificial sweeteners by the sweet taste receptor, T1R2-T1R3, expressed in the enteroendocrine L-cell regulates SGLT1 expression in neighboring absorptive enterocytes. However, the mechanism by which sugar sensing by the enteroendocrine cell is communicated to the absorptive enterocytes is not known. Here, we show that glucagon-like peptide-2 (GLP-2) secreted from the enteroendocrine cell in response to luminal sugars regulates SGLT1 mRNA and protein expression in absorptive enterocytes, via the enteric neurons. Glucose and artificial sweeteners induced secretion of GLP-2 from mouse small intestine, which was inhibited by the sweet-taste receptor inhibitor, gurmarin. In wild type mice there was an increase in sugar-induced SGLT1 mRNA and protein abundance that was not observed in GLP-2 receptor knockout mice. GLP-2 receptor is expressed in enteric neurons, and not in absorptive enterocytes ruling out a paracrine effect of GLP-2. Electric field stimulation of the intestine resulted in upregulation of SGLT1 expression that was abolished by the nerve blocking agent tetrodotoxin. We conclude that GLP-2 and the enteric nervous system are components of the enteroendocrine-absorptive enterocyte communication pathway regulating intestinal glucose transport.

## Introduction

The Na^+^/glucose cotransporter 1, is expressed on the brush border membrane of absorptive enterocytes, and is the major route for transport of dietary glucose across the lumen of the intestine (1, 2). Absorption of glucose via SGLT1 is maximal in proximal small intestine. It has been long recognized that the activity and expression of SGLT1 is upregulated in response to increased intake of dietary carbohydrates or luminal monosaccharides (3, 4). The gut epithelium can sense luminal sugars and artificial sweetens via the G-protein coupled sweet taste receptor, T1R2-T1R3, expressed in enteroendocrine cells to modulate its Na^+^- dependent glucose absorptive capacity (5–7). Short-term intravenous or serosal administration of GLP-2 leads to an increase in the expression of SGLT1, maximal rate of Na^+^-dependent glucose transport and an associated enhancement in blood glucose concentration (8–11). Moreover, increased levels of intracellular cAMP in absorptive enterocytes augment SGLT1 expression by enhancing the half-life of SGLT1 mRNA (12, 13), suggesting the involvement of this second messenger in the SGLT1 regulatory pathway. However, the complete endocrine cell-absorptive enterocyte communication pathway regulating intestinal Na^+^/glucose cotransport is not known.

Expression and activity of SGLT1 are significantly increased in the intestine of patients with type 2 diabetes (14) and obesity (15, 16). However, the reason for this remains unknown. Elucidating the pathway underlying regulation of SGLT1 will further our understanding of deregulation of this process in pathology. In this study our goal was to determine the mechanism by which enteroendocrine sensing of sugar regulates glucose absorptive capacity of absorptive enterocytes, and to investigate the possible role of GLP-2 in this process. Considering that the GLP-2 receptor is expressed in enteric neurons, and not in absorptive enterocytes (17, 18), we present several lines of evidence to demonstrate that GLP-2 regulates SGLT1 mRNA and protein abundance in absorptive enterocytes via a neuro-paracrine pathway. Moreover, we show that the GLP-2 dependent pathway underlying regulation of SGLT1 expression is distinct from GLP-2 induced intestinal growth.

## Materials and methods

### Reagents

All reagents were obtained from Sigma Aldrich (Poole, Dorset, UK) or Fisher Scientific (Loughborough, UK). 8-bromo-cAMP (8-Br-cAMP, B5386, Sigma) was prepared fresh immediately before use. Pituitary adenylate cyclase-activating polypeptide-38 (PACAP, A1439, Sigma), the dominant peptide in all tissues examined, was used in these experiments. Vasoactive intestinal peptide (VIP, V6130, Sigma), PACAP, corticotrophin-releasing hormone (CRH, C3042, Sigma) and calcitonin gene-related peptide (CGRP, C0168, Sigma) were dissolved in 175 mM acetic acid and substance P (S6883, Sigma) in 50 mM acetic acid with 1% (*w*/*v*) bovine serum albumin (A8806, Sigma). Tetrodotoxin (TTX, NA-120, Enzo Life Sciences, Exeter, UK), dissolved at 1 mg/ml in 1% (*v*/*v*) acetic acid, was stored at −20°C and thawed immediately before use. Peptides were stored in aliquots at −80°C and defrosted immediately before use.

### Mice, diets, and tissue collection

The generation of the C57BL/6 Glp2r^−/−^ mice has been reported previously (19). Two male and 2 female Glp2r^+/−^ mice were obtained from Dr. Drucker's laboratory, Toronto, Ontario. They were bred at the licensed breeding facilities at the University of Liverpool Biomedical Services Unit, to produce GLP-2 receptor knockout (Glp2r^−/−^) mice and wild type offspring. Both male and female mice aged between 8 and 10 weeks were used for assessing the effect of GLP-2 receptor deletion on carbohydrate-induced regulation of SGLT1 expression. For all other experiments, male C57BL/6 mice, aged 8 week, purchased from Charles River Laboratories (Margate, Kent, UK), were utilized. All mice had access to water and food and were housed in standard tube cages with automatically controlled temperature and humidity and a 12:12 h light-dark cycle. The amount of daily food intake was similar across diets and genotype (Table S1). All mice were killed by cervical dislocation in accordance with the UK Animals (Scientific Procedures) Act, 1986 and with guidelines set out by the University of Liverpool Ethics Committee and in line with UK Home Office schedule 1 regulations. Depending on the experiment, mice were maintained either on a 40% carbohydrate (CHO)-containing diet (Testdiet #5755, energy 3.70 kcal/g, Purina Mills, Richmond, IN), or standard chow (61.8% CHO, energy 3.60 kcal/g; Special Diet Services, Witham, Essex, UK). Mice used for low/high-CHO feeding were initially kept on the 40% CHO-containing diet before switching to either a low-CHO (1.9% CHO, Testdiet #590N, energy 3.86 kcal/g,) or a high-CHO diet (69.9% CHO, Testdiet #5810, energy 3.77 kcal/g). We have previously demonstrated that SGLT1 expression is upregulated when the CHO (sucrose) content of the diet exceeds 50% (7, 20). The 5-day feeding period was selected to cover intestinal turnover from crypt to villus taking place 4–5 days (21) and 1 day dietary trial was chosen to determine if any increase occurs in existing enterocytes. All diets were isocaloric. To consider the influence of circadian periodicity on SGLT1 expression (22), mice were always killed at 10 am and diets were switched at selected times to ensure that the period of low/high CHO feeding always terminated at 10 am. Immediately after sacrifice, the entire small intestine was removed and the lumen flushed with ice cold 0.9% (*w*/*v*) saline and separated into three equal regions designated proximal, mid and distal. Sections of tissue (1 cm) from the proximal part of each region were fixed for immunohistochemical determinations, or frozen in liquid nitrogen for RNA analysis, with the remaining intestinal tissue being used for brush border membrane vesicle (BBMV) preparations. Tissue (1–2 cm) from C57BL/6 wild-type mice, maintained on chow, was flushed with saline, opened longitudinally and serosa removed by gentle scrapping as reported previously (23). These freshly prepared tissues were employed for gut hormone secretion assays. Hematoxylin (H) and eosin (E) staining confirmed the intactness of the villi with the serosa removed. Proximal intestine, from C57BL/6 wild-type mice maintained on the 40% CHO-containing diet, were used for electrical field stimulation studies.

### Preparation of brush-border membrane vesicles

Brush border membrane vesicles (BBMV) were isolated from intestinal tissues based on the procedure described by Shirazi-Beechey et al. (24) with modifications outlined by Dyer et al. (5). All steps were carried out at 4°C. Tissues were thawed in a buffer solution [100 mM mannitol, 2 mM HEPES/Tris pH 7.1 (BP310, Fisher) with protease inhibitors, 0.5 mM dithiothreitol (D9779, Sigma), 0.2 mM benzamidine (B6506, Sigma), and 0.2 mM phenolmethylsulfonyl fluoride (P7626, Sigma)], cut into small pieces and vibrated for 1 min at speed 5 using a FUNDAMIX vibro-mixer (Dr. Mueller AG, Maennedorf, Switzerland) in order to free epithelial cells. Subsequently, the suspension was filtered through a Büchner funnel to remove any muscle and connective tissues. The filtrate was then homogenized using a Polytron (Ystral, Reading, Berkshire, UK) for 30 s at speed 5. Next, MgCl_2_ was added to the resulting homogenate to a final concentration of 10 mM and the solution stirred on ice for 20 min. The suspension was then centrifuged for 10 min at 3,000 × g (SS34 rotor, Sorvell, UK) and the resulting supernatant was spun for 30 min at 30,000 × g. The pellet was suspended in buffer (100 mM mannitol, 0.1 mM MgSO_4_, and 20 mM HEPES/Tris pH 7.1) and homogenized with 10 strokes of a Potter Elvehjem Teflon hand-held homogenizer before centrifuging for 30 min at 30,000 × g. The final pellet was re-suspended in an isotonic buffer solution (buffer 3, 300 mM mannitol, 0.1 mM MgSO_4_, and 20 mM HEPES/Tris pH 7.4) and homogenized by passing through a 27-gauge needle several times. The protein concentration in the BBMV was estimated by its ability to bind Coomassie blue according to the Bio-Rad assay technique (Bio-Rad, Hemel Hempstead, UK). Porcine γ-globulin (G-2512, Sigma) was used as the standard. In preparation for western blot analysis, aliquots of freshly prepared BBMV were diluted with sample buffer [62.5 mM Tris/HCl pH 6.8, 10% (*v*/*v*) glycerol, 2% (*w*/*v*) SDS, 0.05% (*v*/*v*) β-mercaptoethanol (M-7154, Sigma), 0.05% (*w*/*v*) bromophenol blue (035730, BDH)] and stored at −20°C until use. The remaining BBMV were either diluted 1:100 in buffer 3 and stored at −20°C for enzyme activity determination or were used immediately for glucose uptake studies.

### Immunohistochemistry

Mouse small intestinal tissues, removed from control and Glp2r^−/−^ mice and used for single or triple immunohistochemistry were processed following protocols published previously (7, 20). Small intestinal sheets were fixed for 4 h in 4% paraformaldehyde (P/0840/53, Fisher) and then placed in 20% (w/v) sucrose (84100, Fluka, Gillingham, Dorset, UK) in PBS overnight. Subsequently, tissue samples were gelatin embedded [7.5% (*w/v*) gelatin G1890, Sigma, 15% (*w/v*) sucrose] and frozen in liquid nitrogen cooled isopentane and then kept at −80°C until use. Tissue sections (10 μm thick) were sectioned on a cryostat (Leica, CM 1900UV-1-1, Milton Keynes, Buckinghamshire, UK), thaw mounted onto polylysine coated slides and washed five times for 5 min each in PBS. Slides were then incubated for 1 h in blocking solution. For procedures using antibodies to T1R2 and T1R3 the blocking solution consisted of 5% (*w*/*v*) sucrose, 3% (*w*/*v*) bovine serum albumin (A9647, Sigma), 2% (*v*/*v*) donkey serum, and 0.1% (*w*/*v*) sodium azide in PBS, whereas the blocking solution for procedures using antibodies to VPAC1, VPAC2 (VIP/PACAP receptors 1 & 2) and GLP-2, consisted of 3% (*w*/*v*) bovine serum albumin, 2% (*v*/*v*) donkey serum, and 0.1% (*w/v*) sodium azide in PBS. Tissues were incubated at 4°C overnight with primary antibodies. Antibodies to T1R2 (1:750, sc-22456), T1R3 (1:750, sc-50353), GLP-2 (1:100, sc-7781), VPAC1 (1:100, sc-15958), and VPAC2 (1:100, sc-15961), were purchased from Santa Cruz Biotechnology, INC., Santa Cruz, CA, USA. The composition of the buffer containing antibodies (primary and secondary) was 2.5% (*v/v*) donkey serum, 0.25% (*w/v*) NaN_3_ and 0.2% (*v/v*) triton X-100 (H5141, Sigma) in PBS. For triple immunohistochemistry, two serial tissue sections were incubated with primary antibodies. One section was incubated with two primary antibodies raised in different species whilst the adjacent section was incubated with the third primary antibody. Following incubation with primary antibodies, slides were washed with PBS before incubating with secondary antibodies. Secondary antibodies were either cyanine 3-conjugated anti-goat (705-165-147) or anti-rabbit (711-165-152), raised in donkey, fluorescein isothiocyanate-conjugated anti-rabbit raised in donkey (711-095-152) purchased from Stratech Scientific Ltd (Suffolk, UK), or cyanine 3-conjugated anti-mouse raised in donkey or goat (715-165-151, Jacksons ImmunoResearch Europe Ltd., Cambridge, UK). All secondary antibodies were used at a dilution of 1:500. Finally, the slides were washed with PBS three times for 5 min and mounted in Vectashield hard set mounting media with DAPI (H-1500, Vector Laboratories, Burlington, CA, USA). The immunostaining was visualized using an epifluorescence microscope (Nikon, UK) and images were captured with a Hamamatsu digital camera (C4742-95). Images were merged using Imaging Products Laboratory software (BioVision Technologies, Exton, PA, USA).

### Gut hormone secretion studies

Segments of mouse intestine (~2 cm) were prepared and treated as previously described (25). Briefly, freshly removed 2 cm segments, with the serosa removed, were incubated at 37°C, 5% CO_2_, in incubation media [500 μl, Dulbecco's modified Eagle's medium (5.55 mM glucose, D5546, Sigma) with 10% (*v*/*v*) Fetal Bovine Serum (FBS, EU-000-F, Sera Labs International, UK), 2 mM L-glutamine (G7513, Sigma), 100 U/ml penicillin/ 100 μg/ml streptomycin (P0781, Sigma), 20 μl/ml dipeptidyl peptidase IV inhibitor (DPP4-010, Millipore)], gassed with Carbogen (95% CO_2_, 5% O_2_) and supplemented with appropriate concentrations of test agents: 10–200 mM D-glucose (10117, VWR, Leicestershire, UK), mannitol (M9647, Sigma), or artificial sweetener (10 mM, gift from Pancosma, SA, Switzerland). Control tissues were maintained simultaneously in incubation media. For treatments with gurmarin (the inhibitor of rodent T1R3) (26), tissues were first pre-incubated with 5 μg/ml gurmarin as determined before (23, 25) for 30 min followed by incubation in media containing both gurmarin and test agents. After 1 h, incubation media were collected, centrifuged to remove cell debris, and stored at −80°C. Secretion of GLP-2 was determined using a commercial enzyme immunoassay kit (EK-028-14, Phoenix Pharmaceuticals, Phoenix Europe GmbH, Karlsruhe, Germany), following the manufacturers' instructions. The measurements were within the linear part of the standard curve.

In order to ensure that GLP-2 secretion was not biased by L-cell distribution along the length of the small intestine, parallel longitudinal gut sections for individual mice were used for each test condition. Cellular and tissue integrity were assessed histologically and by measuring the levels of lactate dehydrogenase, a cytoplasmic marker, as described previously (23). Standard curves were constructed using GraphPad Prism 5 (GraphPad Software).

### Electrical field stimulation (EFS)

This procedure was used as described previously (27), with modifications designed for stimulating enteric neurons. Freshly removed intact mouse proximal intestinal tissues maintained in oxygenated (Carbogen) Krebs-Henseleit buffer [110 mM NaCl (S/3160/65, Fisher), 4.7 mM KCl (P/4280/60, Fisher), 3 mM CaCl_2_ (C3306, Sigma), 1.2 mM MgCl_2_, 25 mM NaHCO_2_ (10247, VWR), 1.15 mM NaH_2_PO_4_ (10245, VWR), 5.55 mM D-glucose, 4.9 mM NaPyruvate (P5280, Sigma), 2.7 mM NaFumarate (F1506, Sigma), 4.9 mM NaGlutamate (G1626, Sigma)] at 37°C were flushed with Krebs-Henseleit buffer and divided into 3 cm loops. Each loop was tied at one end with a weight attached in order for the tissue to be kept vertical. The tissue was then placed into a tissue bath containing 10 ml of Krebs-Henseleit buffer, fitted with a water jacket maintained at 37°C. Tissues were allowed to equilibrate in the bath for 20 min. Electric stimulation of the tissue was achieved using 0.5 mm diameter platinum wire electrodes, with one electrode placed inside the intestinal loop at ~3 mm to the luminal membrane and the other outside the loop at the same distance. Electrical current was applied using square wave pulses employing a Grass S44 stimulator (Natus Neurology Incorporated, Grass Products, Warwick PI, USA). The frequency was 20 Hz with duration of 0.3 ms, and delay of 1 ms using a voltage of 50 V (5 mA, measured using Keithley 6485 Instruments, Tektronix UL Ltd. Berkshire, UK). Three sets of tissue loops were used per experiment. The tissue loops which were not stimulated served as controls. One set of tissue loops that did receive EFS were preincubated with 10 μM TTX for 15 min before stimulation in TTX-containing buffer. Immediately following EFS a small section of tissue (0.5 cm) was used for mRNA isolation and the remainder of the tissue for BBMV preparation. In all three sets of tissues, control and stimulated ± TTX, immunohistochemistry showed that tissue integrity was retained and that SGLT1 expression had a similar profile; being expressed on the luminal membrane of villus enterocytes with no expression in the crypt as shown before (20, 25, 28) (data not shown).

### Cell culture

The small intestinal enterocytic cell line Caco-2/TC7 (Caco-2) cells were maintained at 37°C and 5% CO_2_ in Dulbecco's modified Eagles' medium (D6546, Sigma), supplemented with 10% (*v*/*v*) fetal bovine serum, 1% (*v*/*v*) non-essential amino acid solution (M7145, Sigma), L-glutamine (2 mM), and penicillin/streptomycin (100 U/ml; 100 μg/ml) (basal medium). Six well plates were seeded with Caco-2 cells at 100,000 cells/cm^2^ and grown to confluency (~7 d) before being incubated in media containing either 0.5 mM 8-Br-cAMP, as described before (5) or a range of concentrations of VIP, PACAP, CRH, CGRP, or Substance P. Control wells were treated with basal medium only. Equal amounts of solvents, acetic acid or acetic acid with 1% (*w*/*v*) bovine serum albumin, were included in corresponding control media. The time course of the response was determined to establish that 24 h incubation period was the shortest period to give a reliable and significant difference in SGLT1 expression (data not shown). Cells were harvested after 24 h. Cell pellets were collected after centrifugation and frozen at −80°C until used.

### Preparation of post-nuclear membranes

Post-nuclear membranes (PNM) were isolated as described previously (23). Caco-2 cells grown as described above (under 3.7 cell culture) were harvested and cell pellets were collected after centrifugation and frozen at −80°C until used. Caco-2 cell pellets were thawed in buffer [100 mM mannitol, 2 mM HEPES/Tris pH 7.1, 50X complete protease inhibitor cocktail (11836145001, Roche, Sigma)] and homogenized using a Polytron (10 s on setting 5) and centrifuged at 1,000 g for 10 min at 4°C, using a swing-out rotor (HB6, Sovall). The resulting supernatant was centrifuged at 30,000 g for 30 min at 4°C with the resulting pellet re-suspended in 0.5 ml buffer and transferred to a sterile Eppendorf tube and centrifuged at 30,000 g for 30 min at 4°C. The pellet was re-suspended in a buffer consisting of 300 mM mannitol, 20 mM Hepes/tris pH 7.4, 0.1 mM MgSO_4_, and homogenized by passing through a 27G needle several times.

### Western blotting

BBMV were prepared as previously described (24). The abundance of SGLT1 protein in BBMV was determined by western blotting using our well characterized antibody to SGLT1 (5, 7, 20, 29). The antibody to SGLT1 was raised in rabbits (custom synthesis) to a recombinant protein corresponding to amino acids 554-640 of rabbit SGLT1, sharing 81.6% homology to the murine SGLT1 sequence. Protein components of BBMV and PNM were separated by SDS-polyacrylamide gel electrophoresis on 8% (*w*/*v*) polyacrylamide mini gels, containing 0.1% (*w*/*v*) SDS, and electrotransferred to PVDF membrane (1620264, Immun-Blot, Bio-Rad Laboratories Ltd. Hemel Hempstead, UK). Membranes were blocked by incubating for 1 h at room temperature in PBS-TM buffer (PBS containing 0.5% (*w*/*v*) non-fat dried milk (LP0031,Oxoid, UK), and 0.1% (*v*/*v*) Tween-20 (20605, Fisher) before incubation with SGLT1 antibody diluted 1:5,000 in PBS-TM. For the determination of SGLT1 protein abundance in PNM, blocking with 5% (*w*/*v*) milk and an overnight incubation at 4°C were used. Immuno-reactive bands were detected by incubation for 1 h with affinity purified horseradish peroxidase-linked anti-rabbit secondary antibody (P0217, DAKO Ltd, Cambridge, UK) diluted 1:2,000 in PBS-TM. Protein bands were visualized with WEST-one™ western blot detection system (16031, iNtRON Biotechnology, Chembio Ltd., Hertfordshire, UK) and Bio-Max Light Chemiluminescence Film (Z373508, Sigma). Scanning densitometry was performed using Total Lab (TotalLab, Newcastle-upon-Tyne, UK). Membranes were stripped by 3 × 10 min washes in 137 mM NaCl, 20 mM glycine/HCl (pH 2.5) and re-probed with a monoclonal antibody to β-actin (clone AC-15, Sigma Aldrich) used as loading controls. Blocking solution consisted of 5% (*w*/*v*) skimmed milk powder in PBS-TE (PBS, 0.1% (*v*/*v*) Triton X-100, 0.1 mM EDTA). Incubation and washing buffers were also PBS-TE. Horseradish peroxidase-linked anti-rabbit secondary antibody (P0447, DAKO Ltd) diluted 1:2,000 in PBS-TE was used, and visualized as above.

### Measurement of Na^+^-dependent glucose uptake in BBMV

Na^+^-dependent glucose uptake into BBMV was measured as previously described (20, 29). The uptake of D-glucose was initiated by the addition of 100 μl of incubation medium (100 mM NaSCN [or KSCN], 100 mM mannitol, 20 mM HEPES/Tris [pH 7.4], 0.1 mM MgSO_4_, 0.02% (*w*/*v*) NaN_3_, and 0.1 mM [U-^14^C]-D-glucose [10.6 GBq/mmol, NEC043X001MC, Perkin Elmer, Seer Green, Bucks, UK]) to BBMV (100 μg protein) at 37°C. The reaction was stopped after 3 s by the addition of 1 ml of an iso-osmolar ice-cold stop buffer [150 mM KCl 20 mM HEPES/Tris (pH 7.4), 0.1 mM MgSO_4_, 0.02% (*w*/*v*) NaN_3_, and 0.1 mM phloridzin (274313, Sigma)]. Aliquots (0.9 ml) of the reaction mixture were removed and filtered under vacuum through a 0.22 μm pore cellulose acetate/nitrate filter (GSTF02500, Millipore, Hertfordshire, UK). The filter was washed with 5 × 1 ml of ice-cold stop buffer, placed in a vial containing 4 ml of scintillation fluid (Scintisafe 3, SC/9205/21, Fisher Scientific, UK) and the radioactivity retained on the filter was measured using a Tri-Carb 2910TR Liquid Scintillation Analyzer (PerkinElmer, Bucks, UK). All uptakes were measured in triplicate. Suitable substrate concentration, and kinetic parameters of glucose uptake were assessed previously and reported in our publications. Na^+^-dependent glucose uptake is determined by subtracting the glucose uptake in the presence of K^+^ from that of Na^+^.

### Morphometry

Morphometric analysis was performed as previously described (20). Ten micrometer thick sections were exposed to tap water for 1 min, transferred to Mayer's Haemalum (3.3 mM Mayer's Haemalum-haematoxylin, 1 mM sodium iodate, 0.42 mM potassium alum; mhs16, Sigma) for 1 min and washed gently with running tap water for 5 min. They were stained with eosin Y solution [1% (*w*/*v*) eosin aqueous; hs250, HD Supplies, Buckingham, Bucks, UK] for 30 s and subsequently dehydrated by stepwise washing in 70% ethanol (*v*/*v*) for 2 × 1-min, absolute ethanol for 2 × 1-min, and xylene for 3 × 1-min, before mounting with D.P.X. neutral mounting medium (317616, Sigma).

Digital images were captured with an Eclipse E400 microscope and DXM 1200 digital camera (Nikon, Kingston upon Thames, Surrey, UK), analyzed using ImageJ software (Wayne Rasband, US National Institutes of Health, Bethesda, MD) and calibrated using a 100 μm gradient slide. The crypt depth and the villus height were measured as the average distance from crypt base to crypt-villus junction and villus base to villus tip, respectively. The villus height and the crypt depth measurements were taken from an average of 16 well oriented crypt-villus units. A minimum of three images were captured per section at a minimum of 5 sections apart. All images were captured under the same conditions with care taken to ensure that the same villus was not counted twice.

### RNA isolation and quantitative real-time PCR (qPCR)

Expression of SGLT1 and VPAC1/2 mRNA was assessed by quantitative PCR as described before (23). RNA was isolated with the peqGOLD total RNA isolation kit with on-column DNase 1 digestion (12-6834-02, PEQLab, Hampshire, UK), and was used as template for first-strand cDNA synthesis using Superscript III reverse transcriptase (18080-044, Thermo Fisher, UK) and random hexamer primers (SO142, Fisher). cDNA was purified using QiaQuick PCR purification kit (28106, Qiagen, Crawley, UK) and qPCR assays were performed using 25 ng cDNA as template per 25 μl reaction containing SYBR Green JumpStart Taq ReadyMix for qPCR (S4438, Sigma Aldrich) and 900 nM of each primer. PCR cycling was performed as follows: initial denaturation at 95°C for 2 min followed by 30–40 cycles of 95°C for 15 s, 60°C, for 60 s in triplicate using a Rotorgene 3000 (Qiagen, Crawley, UK). Relative abundance was calculated using RG-3000 comparative quantification software with RNA polymerase IIA (POLR2A) as control (see Table S2 for primer sequences).

### Statistics

Commercial software (Graphpad Prism 5) was used for statistical analysis. D'Agostino & Pearson omnibus and Shapiro-Wilk normality tests were used to confirm that continuous variables were normally distributed. Comparison between groups was performed using Student's unpaired *t*-test and one-way ANOVA as appropriate. Dunnett's or Holm-Sidak's multiple comparison post-test was used to determine differences between groups after identification of differences by ANOVA. The level of statistical significance was set at *P* < 0.05.

## Results

### Regulation of SGLT1 expression in wild-type (WT) and GLP-2 receptor knockout mice in response to dietary carbohydrate

**Figure 1:**
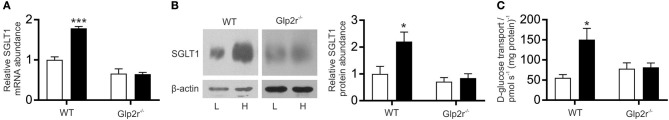
SGLT1 expression in proximal small intestine of wild-type and Glp2r^−/−^ mice in response to consumption of varied levels of dietary carbohydrate. Wild-type (WT) and Glp2 receptor knockout (Glp2r^−/−^) mice were fed low (L, □) or high (H, ■) carbohydrate diets as described in methods. **(A)** Steady state levels of SGLT1 mRNA abundance as determined by qPCR. (**B** *left*) SGLT1 protein abundance in brush-border membrane vesicles (BBMV) isolated from proximal small intestine measured by western blot analysis. (**B** *right*) densitometric analysis of western blots of SGLT1 protein abundance normalized to that of β-actin. **(C)** SGLT1-mediated glucose uptake determined by Na^+^-dependent D-[U^14^C] glucose uptake into same population of BBMV used in B, measured as pmol s^−1^ (mg protein)^−1^. All values are expressed relative to SGLT1 expression in the proximal small intestine of wild-type mice on low-carbohydrate diets for 5 d, as means ± SEM. Data were generated in triplicate, with *n* = 6–8 animals in each group. Statistically significance determined by Student's unpaired two-tailed *t*-test are indicated by *P < 0.05; ***P < 0.001.

Wild type and GLP-2 receptor knockout mice were kept on diets of different carbohydrate content as we described previously (7) for 5 days before measuring intestinal expression of SGLT1. In wild type mice kept on a high- carbohydrate (70% sucrose) diet, SGLT1 mRNA abundance was 2.1-fold higher (*P* < 0.0001) than in WT mice fed a low carbohydrate (1.9% sucrose) diet (Figure 1A). In contrast, GLP-2 receptor knockout mice demonstrated no differences in SGLT1 mRNA expression. The amount of SGLT1 mRNA in knockout mice maintained on either diet was identical to that in wild-type mice on the low carbohydrate diet, indicating that there is a constitutive pathway, independent of GLP-2 action that maintains a basal expression level of SGLT1, and an inducible pathway dependent on GLP-2.

The abundance and activity of SGLT1 protein in brush BBMV isolated from proximal intestine were assessed by western blotting and Na^+^-dependent glucose uptake (Figures 1B,C). In BBMV from WT mice on the high-carbohydrate diet, there was a 2.2-fold increase (*P* = 0.300) in SGLT1 protein abundance compared to the low-carbohydrate diet, which correlated with a 2.7-fold increase (*P* = 0.0387) in the initial rate of Na^+^-dependent glucose transport into BBMV (Figures 1B,C) [Rates of Na^+^-dependent glucose transport were 150.2 ± 28.2 and 55.5 ± 8.0 pmol s^−1^ (mg protein)^−1^ for two diet groups, respectively]; a similar increase in SGLT1 mRNA abundance and glucose transport was observed in BBMV isolated from the mid small intestine (data not shown). There was also a comparable increase in SGLT1 expression and activity when mice were maintained on low- or high-carbohydrate diets for 1 day, indicating that the increase in SGLT1 occurs in the existing enterocytes. Conversely, GLP-2 receptor knockout mice had similar amounts of intestinal SGLT1 protein and Na^+^-dependent glucose transport when maintained on either the low- or high-carbohydrate diet. Thus, whereas wild-type mice are known to respond to increased dietary carbohydrates with enhanced SGLT1 expression (3, 7), GLP-2 receptor knockout mice did not respond in this manner.

Immunohistochemistry showed that SGLT1 protein was expressed, irrespective of genotype, on the luminal membrane of entire villus enterocytes with no expression in the crypt; the intensity of labeling was higher in the intestine of WT mice maintained on a high carbohydrate diet (Figure S1).

**Figure 2:**
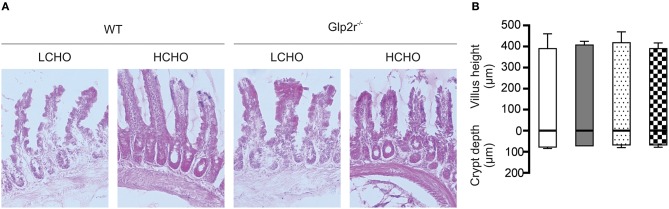
Villus height and crypt depth measurements in wild type and Glp2r^−/−^ mice maintained on low- or high-CHO diets. **(A)** Representative light micrograms of small intestine of wild type and Glp2r^−/−^ mice fed either a low carbohydrate (LCHO) or high-carbohydrate (HCHO) diet as described in the method section. Images represent 10X magnification. **(B)** Morphometric analyses of villus height and crypt depths are shown as histograms, in μm ± SD. Low-CHO-fed wild type (□), High-CHO-fed wild type (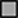), Low-CHO-fed Glp2r^−/−^ (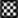), High-CHO-fed Glp2r^−/−^ (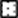); *n* = 4 per group. Statistically significant results were determined using One-way ANOVA with Dunnett's multiple comparison post-test.

Morphometric analysis demonstrated that neither crypt-depth nor villus-height differed in the intestines of WT mice maintained on either a low- or a high-carbohydrate diet (Figure 2), confirming that the observed GLP-2 induced increase in SGLT1 expression is not due to the tropic effect of GLP-2. Moreover, specific activity of sucrase and maltase, two brush border membrane proteins, in the intestine of WT mice maintained either on low- or high-carbohydrate diet were unchanged (Table S3), indicating that the GLP-2 dependent SGLT1 regulatory pathway is distinct from the intestinotrophic effect of GLP-2; the latter influences villus height and the abundance of brush border membrane digestive and absorptive proteins (30).

### Expression of T1R2, T1R3, and GLP-2 in mouse enteroendocrine cells

**Figure 3:**
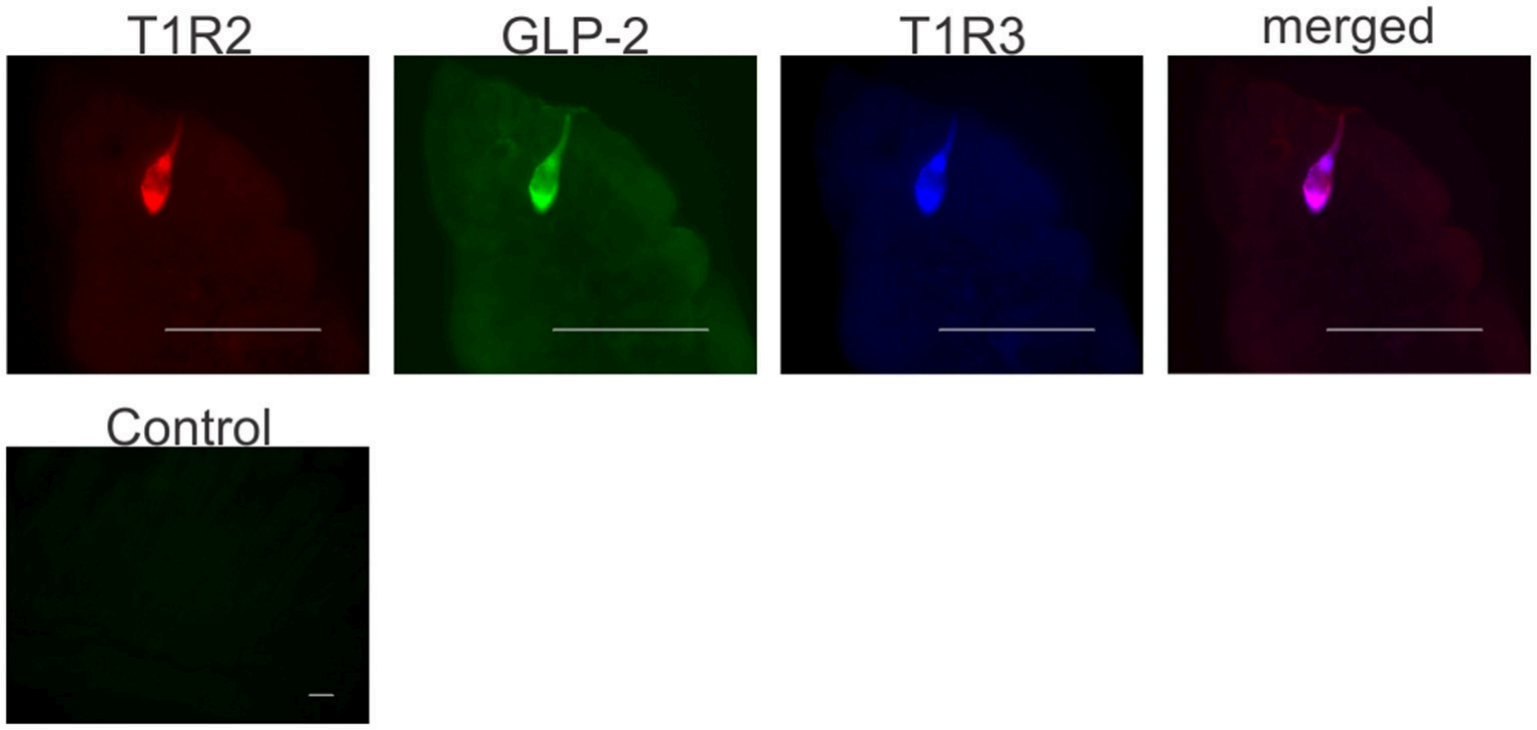
Co-expression of T1R2, T1R3, and GLP-2 in wild-type mice small intestine. Representative image shows expression of T1R2 (red), T1R3 (blue), and GLP-2 (green) in serial sections of wild-type mice small intestine as determined by triple immunohistochemistry. The merged image (purple) shows co-localization of T1R2, T1R3, and GLP-2 in the same enteroendocrine cell. A typical control image showing that pre-incubation of GLP-2 primary antibody with the corresponding peptide antigen blocks the immunoreactive signal. Scale bar = 10 μm.

Having shown GLP-2 involvement in the pathway regulating SGLT1 expression in response to dietary sugars, we assessed the cellular location of T1R2-T1R3 and GLP-2 in mouse proximal intestine. Enteroendocrine cells were identified by the presence of the enteroendocrine cell marker, chromogranin A. Triple immunohistochemistry demonstrated that T1R2, T1R3, and GLP-2 are co-expressed in the same L-enteroendocrine cell (Figure 3). Similar T1R2-T1R3 co-expression was observed in the intestine of the glp2r^−/−^ mice (Figure S2). Pre-incubation of GLP-2 primary antibody with the corresponding peptide antigen blocked the immunoreactive signal, indicating specificity of labeling (Figure 3). T1R2 and T1R3 antibody specificity has been previously validated (7, 20, 25, 28). Approximately 30% of enteroendocrine cells contained GLP-2, with T1R3 and T1R2 being present in 16 and 10% of endocrine cells respectively. The cells that contained T1R2 also possessed GLP-2 and T1R3.

### Glucose and sucralose, but not aspartame, elicit GLP-2 release from mouse small intestine

**Figure 4:**
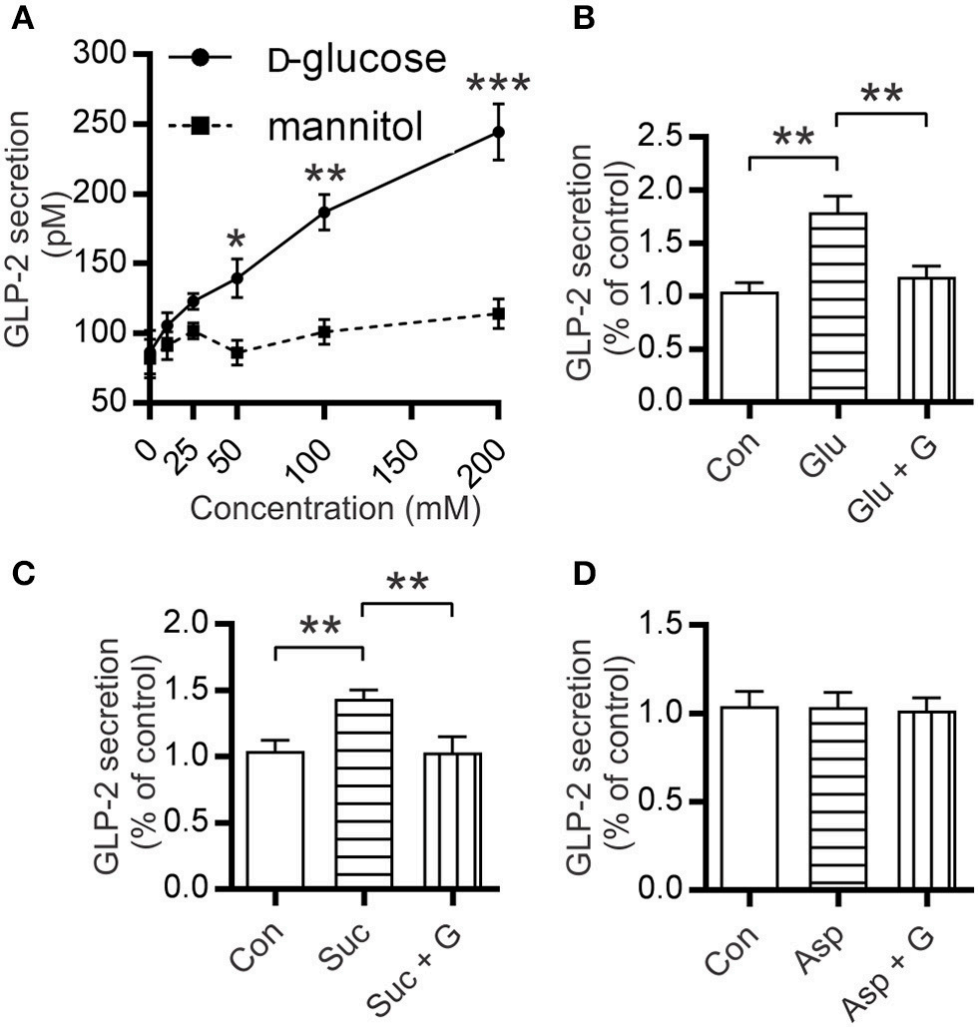
Mouse small intestine secretes GLP-2 in response to glucose and sucralose but not aspartame. **(A)** Secretion levels of GLP-2 by mouse small intestinal tissue sections incubated at 37°C in media supplemented with increasing concentrations of glucose or mannitol. **(B–D)** Measurements of GLP-2 release in media supplemented with either 10 mM of **(B)** glucose (Glu), **(C)** sucralose (Suc), **(D)** aspartame (Asp), ±5 μg/ml gurmarin (*G)*; or untreated (Con); GLP-2 secretion was measured as described in Methods. Data are expressed as means ± SEM. (*n* = 9 per group), where *P*-values are based on unpaired Student's *t*-tests. *P < 0.05, **P < 0.01, and ***P < 0.001.

Segments of mouse proximal small intestine were incubated with increasing concentrations of D-glucose as described previously (25). Results demonstrated a dose-dependent increase in GLP-2 secretion, with 50, 100, and 200 mM D-glucose eliciting 140 ± 14 pM (1.6-fold, *P* = 0.0369), 187 ± 13 pM (2.2-fold, *P* = 0.0051), and 245 ± 20 pM (2.8-fold *P* = 0.0434) compared to control (86.5 ± 15.5 pM), respectively (Figure 4A). To assess the potential effect of media osmolarity, intestinal tissues were also incubated with mannitol (10–200 mM); no GLP-2 secretion above that of the control was observed (Figure 4A). When intestinal tissues were pre-incubated with gurmarin (an inhibitor of mouse T1R3) (26), glucose-induced secretion of GLP-2 was abolished (*P* = 0.0062) (Figure 4B); gurmarin alone had no effect on GLP-2 secretion. In contrast, phloridzin, an inhibitor of SGLT1 had no effect on glucose-induced GLP-2 release ruling out the involvement of SGLT1 as a sensor (data not shown).

To assess the effect of artificial sweeteners on GLP-2 release, mouse intestinal segments were incubated in media supplemented with either 10 mM sucralose or aspartame. Incubation with sucralose resulted in 1.5-fold (*P* = 0.0026) higher secretion of GLP-2 compared to untreated control; this was abolished when intestinal sections were pre-incubated with gurmarin (*P* = 0.0088) (Figure 4C). Aspartame which does not activate mouse T1R2-T1R3 (31) did not induce GLP-2 secretion (Figure 4D). Tissue integrity was assessed by Hematoxylin-Eosin (H&E) staining and determination of lactate dehydrogenase as reported previously (23) (Figure S3).

### Electric field stimulation (EFS) of mouse small intestine enhanced SGLT1 expression

**Figure 5:**
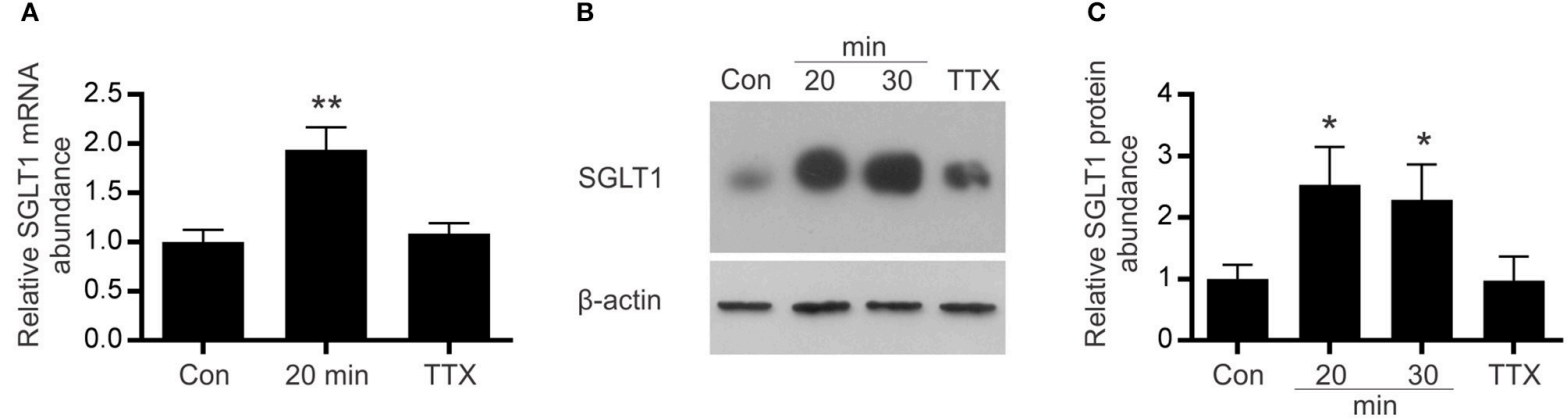
Effect of electric field stimulation on SGLT1 expression. Mouse intestinal loops were stimulated continuously for different periods of time, using frequency of 20 Hz, with 0.3 ms duration, 1 ms delay and 50 V in the absence/presence of TTX or remained unstimulated as described in Methods. **(A)** SGLT1 mRNA abundance as assessed by qPCR, in unstimulated tissue (Con), stimulated tissue (20 min), or tissue stimulated for 20 min in the presence of the 10 μM tetrodotoxin (TTX). **(B)** Abundance of SGLT1 protein, determined by western blot analysis, in BBMV isolated from mouse small intestinal tissue that was either stimulated for 20 or 30 min (20 and 30 min), in the presence of tetrodotoxin (TTX), or left unstimulated (Con). **(C)** Densitometric analysis of western blots normalized to β-actin protein abundance (*n* = 5–9). Data are expressed as means ± SEM. *P*-values are based on unpaired Student's *t*-tests. *P < 0.05; **P < 0.01.

In response to 20 min of EFS, there was a 2-fold (*P* = 0.0037) increase in SGLT1 mRNA levels (and remained the same after 30 min) compared to unstimulated tissue (Figure 5A). A similar increase in SGLT1 protein abundance, 2.3-fold (*P* = 0.0461), was also observed in tissues stimulated for 20 min (Figures 5B,C). This increase in SGLT1 expression was abolished when the tissue was pre-incubated with the neuronal sodium-channel blocker TTX (Figures 5A–C). TTX alone had no effect on SGLT1 expression in unstimulated tissue (data not shown).

### The effect of modulators of intracellular cAMP on SGLT1 expression in Caco-2 cells

A widely used model cell line of intestinal absorptive enterocytes, Caco-2/TC7, was used to assess expression levels of SGLT1 in response to a number of agents known to increase intracellular cAMP. The time course of response to 8-Br-cAMP (a membrane permeable analog of cAMP) was determined in order to establish the shortest time that elicits a significant increase in SGLT1 expression. In response to 0.5 mM 8-Br-cAMP, steady-state levels of SGLT1 mRNA increased 2.3-fold (*P* < 0.001) after 24 h (Figures 5A–C).

**Figure 6:**
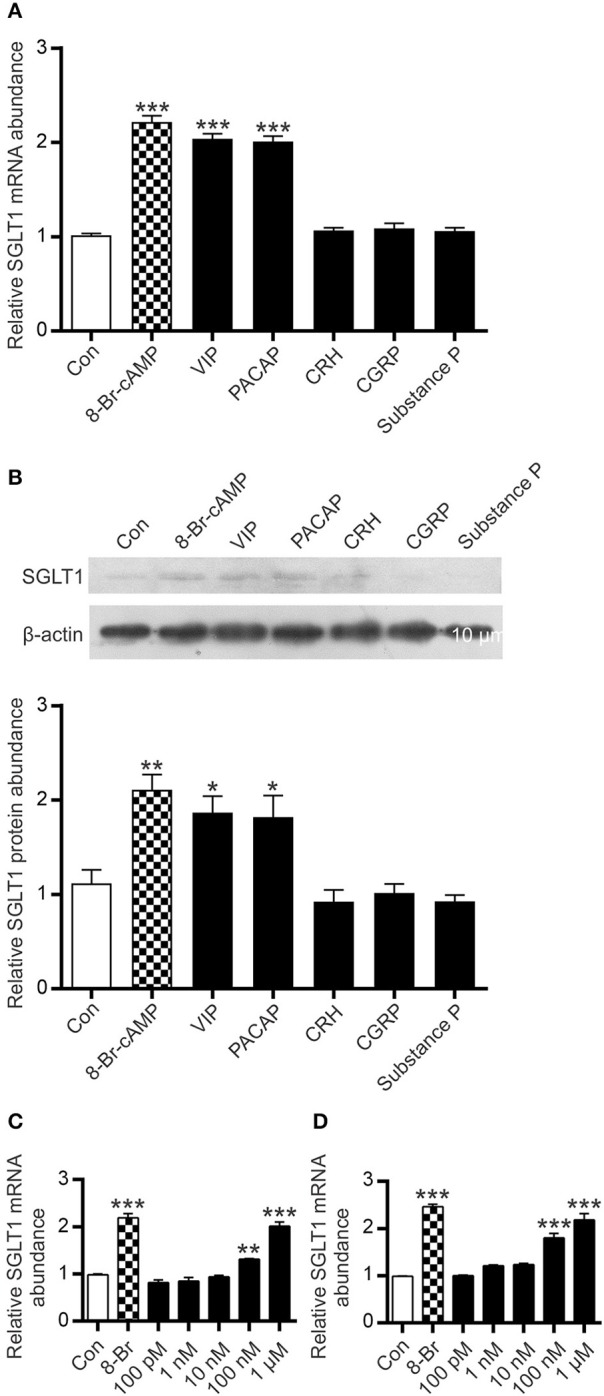
Effect of intracellular cAMP modulators on SGLT1 protein and mRNA expression in Caco-2/TC7 cells. Confluent Caco-2 cells were exposed to either 0.5 mM 8-Br-cAMP (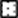), 1μM of each, VIP, PACAP, CRH, CGRP, or Substance P, or maintained in untreated medium (□). **(A)** Expression of SGLT1 mRNA, as determined by qPCR, in Caco-2 cells, *n* = 15. **(B)** Expression of SGLT1 protein abundance, as determined by western blotting, in PNM isolated from Caco-2 cells (upper panel). Densitometric analysis of western blots of SGLT1 protein abundance was normalized to that of β-actin (lower panel), *n* = 3. **(C,D)** SGLT1 mRNA expression measured in response to graded concentrations (100 pM−1 μM) of VIP **(C)** and PACAP **(D)**, *n* = 15. Data are expressed as means ± SEM. *P*-values are based on one-way ANOVA with Dunnett's multiple comparison post-test. *P < 0.05, **P < 0.01, and ***P < 0.001.

Having shown that electric field stimulation of enteric neurons resulted in enhanced expression of SGLT1, we tested several neuropeptides for their effect on SGLT1 mRNA abundance in Caco-2 cells. The principal selection criteria were (i) established stimulatory effect on cAMP accumulation (ii) action via stimulatory GPCR (Gs), and (iii) known expression in intestinal tissue. Accordingly, VIP, PACAP, CRH, CGRP, and substance P were selected. Substance P, while acting principally via Gq, is reported to increase cAMP formation via Gs (32). Cells were exposed for 24 h to a range of concentrations (10 nM−1 μM) of neuropeptides. In response to 100 nM VIP, there was a 1.3-fold increase in SGLT1 mRNA abundance (*P* < 0.01) with a 2-fold increase observed at 1 μM (*P* < 0.001); the latter a similar magnitude of response to that seen after exposure to 0.5 mM 8-Br-cAMP (Figures 6A,C). The effect of PACAP also increased with concentration, with a 1.8-fold increase in SGLT1 mRNA at 100 nM and a 2.2-fold increase at 1 μM (both *P* < 0.001; Figures 6A,D). When Caco-2 cells were incubated with 1 μM VIP and PACAP there was a 1.7- (*P* = 0.0155) and 1.6-fold (*P* = 0.0236) increase in SGLT1 protein abundance a for VIP and PACAP respectively (Figure 6B). In contrast, CRH, CGRP and Substance P had no effect on SGLT1 mRNA and protein expression (Figures 6A,B).

### Vasoactive intestinal polypeptide receptor 1 (VPAC1) is expressed on the basolateral membrane of absorptive enterocytes

**Figure 7:**
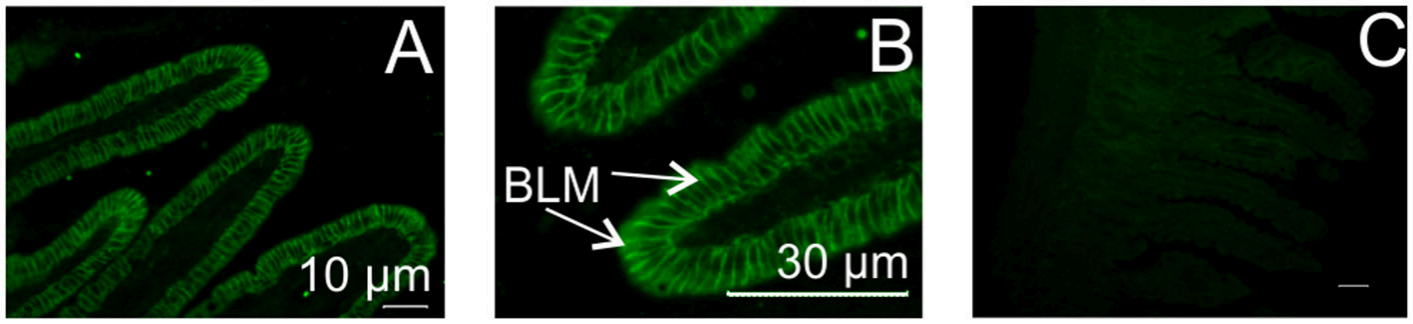
Localization of VIP/PACAP receptor in mouse small intestine. Typical immunofluorescent images showing expression of VPAC1 protein on the basolateral membrane of mouse intestinal enterocytes **(A,B)**. No labeling was observed with omission of the primary antibody (**C**, control). VPAC2 protein was not expressed. BLM, basolateral membrane.

Biological responses induced by VIP or PACAP are triggered by interaction with two receptors, VPAC1 and VPAC2, which are mainly coupled to the stimulatory G-protein, Gs, resulting in stimulation of cellular adenylate cyclase (33). Using PCR, we showed that Caco-2 cells express VPAC1 mRNA, but not VPAC2, while mouse small intestine possesses both VPAC1 and VPAC2 mRNA (data not shown). By immunohistochemistry, we demonstrated that VPAC1 is expressed on the basolateral membrane of mouse absorptive enterocytes (Figures 7A,B); whereas no labeling for VPAC2 was observed on the plasma membrane of any intestinal epithelial cells (data not shown). Pre-incubation of the antibody with the immunizing peptide blocked the labeling, showing antibody specificity (Figure 7C).

## Discussion

SGLT1 is expressed on the apical membrane of enterocytes, and is the major route for absorption of dietary glucose. Sensing of glucose by the sweet taste receptor, T1R2-T1R3, expressed in enteroendocrine L-cells, activates a pathway leading to upregulation of SGLT1 expression in neighboring absorptive enterocytes (7).

In response to luminal glucose, L-enteroendocrine cells secrete the gut hormone, GLP-2 (34). L-cells have been identified throughout the intestinal tract, with the highest numbers observed in the distal intestine (35). However, the proximal intestine has the greater density of GLP-2 receptor cells (36), and shows the largest response to pharmacological stimulation of the GLP-2 receptor (37, 38). GLP-2 is implicated in enhancing SGLT1 expression and intestinal glucose transport (8–11). It exerts it effect by binding to its receptor (GLP-2R).

Here, we show that knocking out the GLP-2 receptor abolishes the ability of mouse intestine to increase SGLT1 expression in response to increased dietary carbohydrate. This supports our hypothesis that GLP-2 is part of the sugar-induced pathway in the intestinal mucosa that regulates SGLT1 expression.

Immunohistochemistry on the intestinal tissues of WT and GLP2r^−/−^ mice maintained on low or high carbohydrate diets demonstrated that in all cases, irrespective of genotype and diet, SGLT1 protein is expressed on the luminal membrane of entire villus enterocytes with no labeling in the crypt. Thus, lack of response to low and high carbohydrate diet in GLP2r^−/−^ is not due to altered profile of SGLT1 protein expression along crypt-villus axis, but due to absence of receptor for GLP-2 required for this gut hormone to bind to, in order to exert its biological effect.

It is now widely recognized that GLP-2 receptor is expressed in the submucosa and myenteric plexus and not in intestinal epithelial cells (17, 18). The enteric neurons send most of their axonal projections to the muscle layers of intestine, while submucosal plexus neurons send the majority of their projections to sub-epithelial regions. The findings that GLP-2 receptor is expressed in the submucosa and myenteric plexus and not in intestinal epithelial cells rule out a direct paracrine effect of GLP-2 on neighboring absorptive enterocytes and proposes a potential role for the enteric nervous system in the regulatory pathway underlying GLP-2 induced upregulation of SGLT1 expression. Neuronal action potential mostly depends on the function of voltage gated sodium channels which are specifically inhibited by TTX (39). We show that pre-treatment of mouse intestinal tissue with TTX abolishes the upregulation of SGLT1 mRNA and protein in response to electric field stimulation, signifying the involvement of enteric nervous system in the regulatory process.

Increased levels of intracellular cAMP in absorptive enterocytes lead to upregulation of SGLT1 expression (12) demonstrating the involvement of this second messenger in the SGLT1 regulatory pathway. It has been shown that a number of cAMP-elevating agents increase SGLT1 expression at levels of mRNA, protein and function (5, 40) and that cAMP-induced post-transcriptional regulation of mRNA stability plays a major role in SGLT1 upregulation.

Our experimental evidence suggests that T1R2-T1R3, expressed in enteroendocrine L-cells, detects luminal glucose concentrations. When above a threshold, glucose activates a signaling pathway involving T1R2-T1R3, the associated G-protein gustducin, and other signaling elements resulting in GLP-2 secretion. The binding of GLP-2 to its receptor on the submucosal plexus elicits a neuronal response (17) which is transmitted to sub-epithelial regions through axonal projections that are in close proximity to the basolateral membrane domain of absorptive enterocytes (41) evoking the release of VIP or PACAP. Binding of VIP and/or PACAP to its receptor, VPAC1, expressed on the basolateral membrane of absorptive enterocytes, enhances intracellular levels of cAMP, thereby increasing SGLT1 expression.

It is noteworthy that VIP is released from the intestine in response to electric field stimulation (42) and that GLP-2 increases enteric neuronal expression of VIP *in vivo* (43).

It has been shown that long term GLP-2 administration results in increased crypt-villus height (44, 45) and that in the small intestine, insulin growth factor 1 (IGF-1) (and likely other growth factors such as ErbB ligands) is an essential mediator of the intestinotrophic action of GLP-2 (30, 46, 47). Assessing the intestinal tissues of WT and IGF-1 knockout mice (tissues kindly provided by Drs. Brubaker and Dubé, University of Toronto) we demonstrated that there were no differences in the expression level of SGLT1 between WT and IGF-1 knockout mice when maintained on a high (61.8%) carbohydrate diet (data not shown), indicating that the GLP-2 dependent pathway underlying regulation of SGLT1 is distinct from GLP-2 induced intestinal growth.

In the intestine of patients with type 2 diabetes, there is enhanced expression of SGLT1 mRNA, protein abundance and Na^+^-dependent D-glucose uptake (14). The increase is independent of the amount of dietary carbohydrate intake, blood glucose or insulin, and is likely to be due to deregulation of the pathway regulating expression of intestinal SGLT1 (14). The ability of the intestine to increase glucose absorption in diabetes further complicates the pathophysiology of this disease. The understanding of the regulatory pathway controlling SGLT1 expression allows identification of targets for controlling the capacity of the gut to absorb glucose. This has attendant promise for preventing and/or treating conditions such as malabsorption, diabetes and obesity.

## Author contributions

SS-B is responsible for conception and design of the research with AM designing and carrying out experiments using Glp2r^−/−^ mice, EFS, gut hormone measurements, functional assays, western blotting and qPCR analyses of SGLT1 expression. MA-R performed all immunohistochemical analyses. SS-B and DJB designed experiments with DJB performing experiments on the effect of neuropeptides, and DMB provided nutritional advice. SS-B and AM analyzed and interpreted the data. SS-B wrote the paper.

### Conflict of interest statement

DMB was an employee of Pancosma SA. The remaining authors declare that the research was conducted in the absence of any commercial or financial relationships that could be construed as a potential conflict of interest.

## References

[1] Shirazi-Beechey SP. Molecular biology of intestinal glucose transport. Nutr Res Rev. (1995) 8:27–41. 10.1079/NRR19950005 19094278

[2] Gorboulev V Schürmann A Vallon V Kipp H Jaschke A Klessen D. Na(+)-D-glucose cotransporter SGLT1 is pivotal for intestinal glucose absorption and glucose-dependent incretin secretion. Diabetes (2012) 61:87–196. 10.2337/db11-1029 22124465 PMC3237647

[3] Diamond JM Karasov WH. Adaptive regulation of intestinal nutrient transporters. Proc Natl Acad Sci USA. (1987) 84:2242–5. 3470788 10.1073/pnas.84.8.2242 PMC304625

[4] Ferraris RP Diamond J. Regulation of intestinal sugar transport. Physiol Rev. (1997) 77:257–302. 9016304 10.1152/physrev.1997.77.1.257

[5] Dyer J Vayro S King TP Shirazi-Beechey SP. Glucose sensing in the intestinal epithelium. Eur J Biochem. (2003) 270:3377–88. 10.1046/j.1432-1033.2003.03721.x 12899695

[6] Dyer J Salmon KS Zibrik L Shirazi-Beechey SP. Expression of sweet taste receptors of the T1R family in the intestinal tract and enteroendocrine cells. Biochem Soc Trans. (2005) 33:302–5. 10.1042/BST0330302 15667333

[7] Margolskee RF Dyer J Kokrashvili Z Salmon KS Ilegems E Daly K. T1R3 and gustducin in gut sense sugars to regulate expression of Na+-glucose cotransporter 1. Proc Natl Acad Sci USA. (2007) 104:15075–80. 10.1073/pnas.0706678104 17724332 PMC1986615

[8] Ramsanahie A Duxbury MS Grikscheit TC Perez A Rhoads DB Gardner-Thorpe J. Effect of GLP-2 on mucosal morphology and SGLT1 expression in tissue-engineered neointestine. Am J Physiol Gastrointest Liver Physiol. (2003) 285:G1345–52. 10.1152/ajpgi.00374.2002 12919941

[9] Sangild PT Tappenden KA Malo C Petersen YM Elnif J Bartholome AL. Glucagon-like peptide 2 stimulates intestinal nutrient absorption in parenterally fed newborn pigs. J Pediatr Gastroenterol Nutr. (2006) 43:160–67. 10.1097/01.mpg.0000228122.82723.1b 16877979

[10] Cheeseman CI. Upregulation of SGLT-1 transport activity in rat jejunum induced by GLP-2 infusion *in vivo*. Am J Physiol Regul Integr Comp Physiol. (1997) 273:R1965–71. 9435650 10.1152/ajpregu.1997.273.6.R1965

[11] Burrin D Guan X Stoll B Petersen YM Sangild PT. Glucagon-like peptide 2: a key link between nutrition and intestinal adaptation in neonates? J Nutr. (2003) 133:3712–6. 10.1093/jn/133.11.3712 14608101

[12] Lee WY Loflin P Clancey CJ Peng H Lever JE. Cyclic nucleotide regulation of Na+/glucose cotransporter (SGLT1) mRNA stability; Interaction of a nucleocytoplasmic protein with a regulatory domain in the 3'-untranslated region critical for stabilization. J Biol Chem. (2000) 275:33998–4008. 10.1074/jbc.M005040200 10950955

[13] Loflin P Lever JE. HuR binds a cyclic nucleotide-dependent, stabilizing domain in the 3' untranslated region of Na(+)/glucose cotransporter (SGLT1) mRNA. FEBS Lett. (2001) 509:267–71. 10.1016/S0014-5793(01)03176-3 11741601

[14] Dyer J Wood IS Palejwala A Ellis A Shirazi-Beechey SP. Expression of monosaccharide transporters in intestine of diabetic humans. Am J Physiol Gastrointest Liver Physiol. (2002) 282:G241–8. 10.1152/ajpgi.00310.2001 11804845

[15] Nguyen NQ Debreceni TL Bambrick JE Chia B Wishart J Deane AM. Accelerated intestinal glucose absorption in morbidly obese humans: relationship to glucose transporters, incretin hormones, and glycemia. J Clin Endocrinol Metab. (2015) 100:968–76. 10.1210/jc.2014-3144 25423571

[16] Wölnerhanssen BK Moran AW Burdyga G Meyer-Gerspach AC Peterli R Manz M. Deregulation of transcription factors controlling intestinal epithelial cell differentiation; a predisposing factor for reduced enteroendocrine cell number in morbidly obese individuals. Sci Rep. (2017) 7:8174. 10.1038/s41598-017-08487-9 28811552 PMC5557953

[17] Bjerknes M Cheng H. Modulation of specific intestinal epithelial progenitors by enteric neurons. Proc Natl Acad Sci USA (2001) 98:12497–502. 10.1073/pnas.211278098 11572941 PMC60082

[18] Pedersen J Pedersen NB Brix SW Grunddal KV Rosenkilde MM Hartmann B The glucagon-like peptide 2 receptor is expressed in enteric neurons and not in the epithelium of the intestine. Peptides (2015) 67:20–8. 10.1016/j.peptides.2015.02.007 25748021

[19] Lee SJ Lee J Li KK Holland D Maughan H Guttman DS. Disruption of the murine Glp2r impairs Paneth cell function and increases susceptibility to small bowel enteritis. Endocrinology (2012) 153:1141–51. 10.1210/en.2011-1954 22253424 PMC3606134

[20] Moran AW Al-Rammahi MA Arora DK Batchelor DJ Coulter EA Ionescu C. Expression of Na^+^/glucose co-transporter 1 (SGLT1) in the intestine of piglets weaned to different concentrations of dietary carbohydrate. Br J Nutr. (2010) 104:647–55. 10.1017/S0007114510000954 20385036

[21] Mayhew TM Mykleburst R Whybrow A Jenkins R. Epithelial integrity, cell death and cell loss in mammalian small intestine. Histol Histopathol. (1999) 14:257–67. 9987670 10.14670/HH-14.257

[22] Balakrishnan A Stearns AT Rounds J Irani J Giuffrida M Rhoads DB. Diurnal rhythmicity in glucose uptake is mediated by temporal periodicity in the expression of the sodium-glucose cotransporter (SGLT1). Surgery (2008) 143:813–8. 10.1016/j.surg.2008.03.018 18549898 PMC2600898

[23] Daly K Al-Rammahi M Moran A Marcello M Ninomiya Y Shirazi-Beechey SP. Sensing of amino acids by the gut-expressed taste receptor, T1R1-T1R3, stimulates CCK secretion. Am J Physiol Gastrointest Liver Physiol. (2013) 304:G271–82. 10.1152/ajpgi.00074.2012 23203156 PMC3566511

[24] Shirazi-Beechey SP Davies AG Tebbutt K Dyer J Ellis A Taylor CJ. Preparation and properties of brush-border membrane vesicles from human small intestine. Gastroenterology (1990) 98:676–85. 10.1016/0016-5085(90)90288-C 2298371

[25] Daly K Al-Rammahi M Arora DK Moran AW Proudman CJ Ninomiya Y. Expression of sweet receptor components in equine small intestine: relevance to intestinal glucose transport. Am J Physiol Regul Integr Comp Physiol. (2012) 303:R199–208. 10.1152/ajpregu.00031.2012 22552794

[26] Ninomyia Y Imoto T Gurmarin inhibition of sweet taste responses in mice. Am J Physiol Regul Integr Comp Physiol. (1995) 268:R1019–25. 10.1152/ajpregu.1995.268.4.R1019 7733384

[27] Davison JS Pearson GT Petersen OH Mouse Pancreatic Acinar Cells: Effects of electric field stimulation on membrane potential and resistance. J Physiol. (1980) 301:295–305. 6251202 10.1113/jphysiol.1980.sp013206 PMC1279399

[28] Batchelor DJ Al-Rammahi M Moran AW Brand JG Li X Haskins M Intestinal sodium/glucose cotransporter-1 (SGLT1) and disaccharidase expression in the domestic dog and cat: two species of different dietary habit. Am J Physiol Regul Integr Comp Physiol. (2011) 300:R67–75. 10.1152/ajpregu.00262.2010 20980625 PMC3023277

[29] Dyer J Hosie KB Shirazi-Beechey SP. Nutrient regulation of human intestinal sugar transporter (SGLT1) expression. Gut (1997) 41:56–9. 9274472 10.1136/gut.41.1.56 PMC1027228

[30] Rowland KJ Brubaker PL. The “cryptic” mechanism of action of glucagon-like peptide 2. Am J Physiol Gastrointest Liver Physiol. (2011) 301:G1–8. 10.1152/ajpgi.00039.2011 21527727

[31] Li X Staszewski L Xu H Durick K Zoller M Adler E. Human receptors for sweet and umami taste. Proc Natl Acad Sci USA. (2002) 99:4692–6. 10.1073/pnas.072090199 11917125 PMC123709

[32] Tuluc F Lai JP Kilpatrick LE Evans DL Douglas SD. Neurokinin 1 receptor isoforms and the control of innate immunity. Trends Immunol. (2009) 30:271–6. 10.1016/j.it.2009.03.006 19427266

[33] Couvineau A Ceraudo E Tan YV Laburthe M. VPAC1 receptor binding site: contribution of photoaffinity labeling approach. Neuropeptides (2010) 44:127–32. 10.1016/j.npep.2009.11.008 20031208

[34] Drucker DJ Yusta B. Physiology and pharmacology of the enteroendocrine hormone glucagon-like peptide-2. Annu Rev Physiol. (2014) 76:561–83. 10.1146/annurev-physiol-021113-170317 24161075

[35] Steinert RE Gerspach AC Gutmann H Asarian L Drewe J Beglinger C. The functional involvement of gut-expressed sweet taste receptors in glucose-stimulated secretion of Glucogon-Like Peptide-1 (GLP-1) and Peptide YY (PYY). Clin Nutr. (2011) 30:524–32. 10.1016/j.clnu.2011.01.007 21324568

[36] Ørskov C Hartmann B Poulsen SS Thulesen J Hare KJ Holst JJ. GLP-2 stimulates colonic growth via KGF, released by subepithelial myofibroblasts with GLP-2 receptors. Regul Pept. (2005) 124:105–12. 10.1016/j.regpep.2004.07.009 15544847

[37] Hare KJ Hartmann B Kissow H Holst JJ Poulsen SS. The intestinotrophic peptide, glp-2, counteracts intestinal atrophy in mice induced by the epidermal growth factor receptor inhibitor, gefitinib. Clin Cancer Res. (2007) 13:5170–5. 10.1158/1078-0432.CCR-07-0574 17785573

[38] Pedersen NB Hjollund KR Johnsen AH Orskov C Rosenkilde MM Hartmann B. Porcine glucagon-like peptide-2: structure, signaling, metabolism and effects. Regul Pept. (2008) 146:310–20. 10.1016/j.regpep.2007.11.003 18164496

[39] Bane V Lehane M Dikshit M O'Riordan A Furey A. Tetrodotoxin: chemistry, toxicity, source, distribution and detection. Toxins (2014) 6:693–755. 10.3390/toxins6020693 24566728 PMC3942760

[40] Peng H Lever JE. Regulation of Na(+)-coupled glucose transport in LLC-PK1 cells. Message stabilization induced by cyclic AMP elevation is accompanied by binding of a M(r) = 48,000 protein to a uridine-rich domain in the 3′-untranslated region. J Biol Chem. (1995) 270:23996–4003. 7592596 10.1074/jbc.270.41.23996

[41] Mills JC Gordon JI. The intestinal stem cell niche: there grows the neighborhood. Proc Natl Acad Sci USA. (2001) 98:12334–6. 10.1073/pnas.231487198 11675485 PMC60050

[42] Gaginella TS O'Dorisio TM Hubel KA. Release of vasoactive intestinal polypeptide by electrical field stimulation of rabbit ileum. Regul Pept. (1981) 2:165–74. 7255769 10.1016/0167-0115(81)90010-0

[43] de Heuvel E Wallace L Sharkey KA Sigalet DL. Glucagon-like peptide 2 induces vasoactive intestinal polypeptide expression in enteric neurons via phophatidylinositol 3-kinase-γ signaling. Am J Physiol Endocrinol Metab. (2012) 303:E994–1005. 10.1152/ajpendo.00291.2012 22895780 PMC3469609

[44] Brubaker PL Izzo A Hill M Drucker DJ Intestinal function in mice with small bowel growth induced by glucagon-like peptide-2. Am J Physiol Endocinol Metab. (1997) 35:E1050–8. 10.1152/ajpendo.1997.272.6.E1050 9227451

[45] Drucker DJ DeForest L Brubaker PL Intestinal response to growth factors administered alone or in combination with human [Gly^2^]glucagon-like peptide 2. Am J Physiol Gastrointest Liver Physiol. (1997) 36:G1252–62. 10.1152/ajpgi.1997.273.6.G1252 9435550

[46] Dubé PE Forse CL Bahrami J Brubaker PL. The essential role of insulin-like growth factor-1 in the intestinal tropic effects of glucagon-like peptide-2 in mice. Gastroenterology (2006) 131:589–605. 10.1053/j.gastro.2006.05.055 16890611

[47] Leen JLS Izzo A Upadhyay C Rowland KJ Dubé PE Gu S Mechanism of action of glucagon-like peptide-2 to increase IGF-1 mRNA in intestinal subepithelial fibroblasts. Endocrinology (2011) 152:436–46. 10.1210/en.2010-0822 21159855 PMC3384785

