# Supplementary material for: Glucagon-Like Peptide-2 and the Enteric Nervous System Are Components of Cell-Cell Communication Pathway Regulating Intestinal Na+/Glucose Co-transport
Source: Front Nutr. 2018 Oct 26;5:101. doi: 10.3389/fnut.2018.00101 (PMC6212479; doi:10.3389/fnut.2018.00101)
Supplement: Supplementary file 1 [file Data_Sheet_1.docx]

Supplementary Material

**Glucose sensing and neuroparacrine pathways underlie regulation of intestinal Na^+^/glucose co-transporter 1, SGLT1.**

Andrew W. Moran, Miran A. Al-Rammahi, Daniel J. Batchelor, David Bravo and Soraya P Shirazi-Beechey^*^

*** Correspondence:** Corresponding Author: Soraya P. Shirazi-Beechey, Epithelial Function and Development Group, Department of Functional and Comparative Genomics, Institute of Integrative Biology, Bioscience Building, University of Liverpool, Liverpool, L69 7ZB, United Kingdom. Tel: +44 151 794 4255. Fax: +44 151 794 4244. Email: [spsb@liverpool.ac.uk](mailto:spsb@liverpool.ac.uk)

# Supplementary Figures and Tables

## Supplementary Figures

##
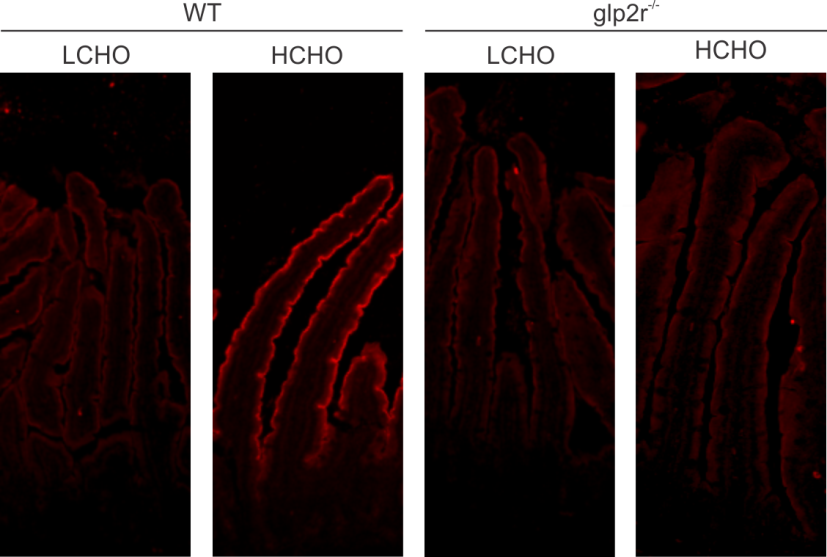


**Supplementary Figure 1.** SGLT1 expression in WT and glp2r^-/-^ mouse proximal small intestine. Immunofluorescent staining of SGLT1 (red) in WT and glp2r^-/-^ mouse small intestine fed either a low- (LCHO) or high-carbohydrate (HCHO) diet. Images taken with 100X magnification.

**
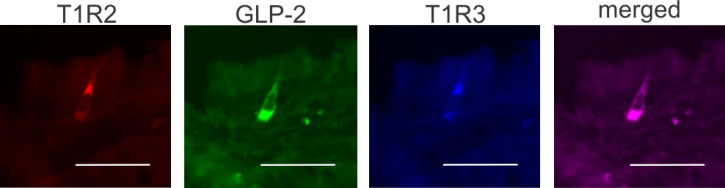
**

**Supplementary Figure 2.** Expression of T1R2, T1R3 and GLP-2 in *glp2r^-/-^* mouse L-endocrine cell. A representative image showing expression of T1R2 (red), T1R3 (blue) and GLP-2 (green) in serial sections of *glp2r^-/-^* mouse small intestine (whether maintained on a low or a high carbohydrate diet) as determined by triple immunohistochemistry. The merged image (purple) shows co-localization of T1R2, T1R3 and GLP-2 in the same enteroendocrine cell. Scale bar = 10 µm. Knocking out the GLP-2 receptor has no effect on expression, abundance and cellular location of T1R2, T1R3 and GLP-2


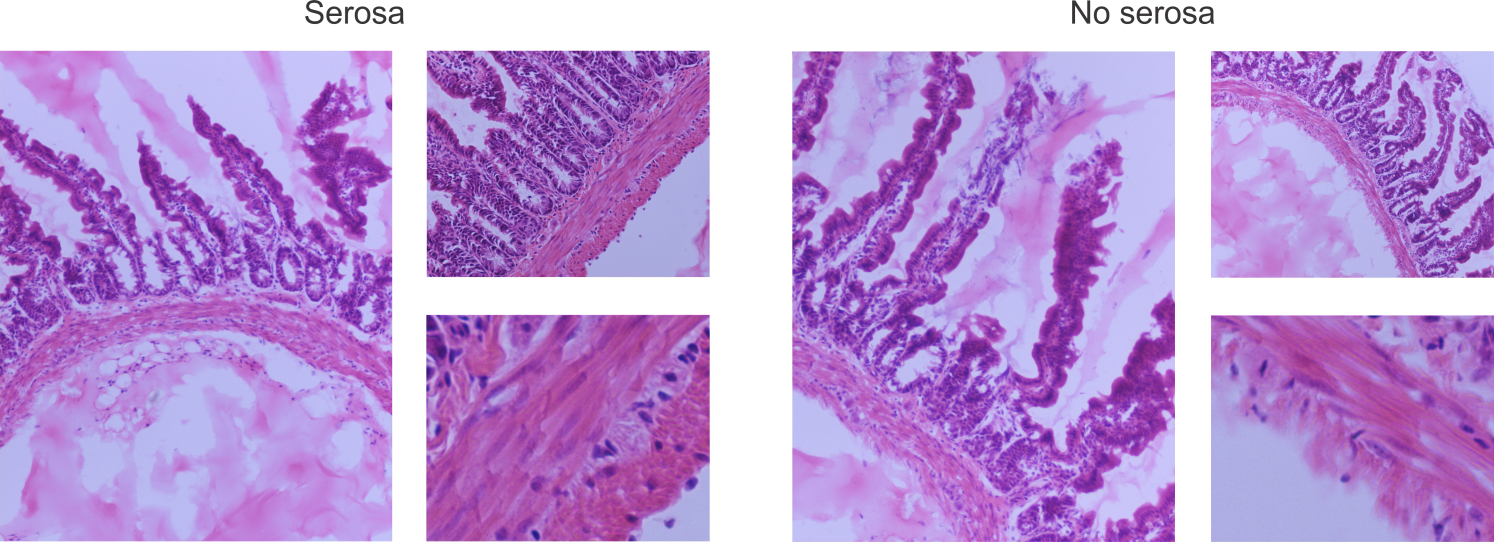


**Supplementary Figure 3**: Hematoxylin-Eosin staining of mouse small intestinal tissues showing tissue intactness after serosa removed. Typical light micrographs showing intactness of proximal small intestine of wild type mice when serosa removed, compared to intact tissue with serosa. Images were obtained at 100-400X magnification.

## Supplementary Tables

**Table S1**: Feed intake of WT and Glp2r^-/-^ mice.

| Feed intake (g / d) | | | |
| --- | --- | --- | --- |
| 5d WT LCHO | 5d WT HCHO | 5d KO LCHO | 5d KO HCHO |
| 3.2 ± 1.1 | 4.2 ± 0.9 | 3.7 ± 0.9 | 4.7 ± 1.5 |

**Table S2**: Primers for human and mouse VPAC1, VPAC2, SGLT1, POLR2A and Villin.

| **Name** | **Sequence** |
| --- | --- |
| Human VPAC_1_ S | GATGTGGGACAACCTCACCT |
| Human VPAC_1_ AS | GAGGGCCAAGTCTTTGATGA |
| Human VPAC_2_ S | CCAGAATGCCGATTTCATCT |
| Human VPAC_2_ AS | AGCTTCCTGAAGAGGCACAG |
| Mouse VPAC1 S | GATGTGGGACAACCTCACCT |
| Mouse VPAC1 AS | ACCGAGCCTCAGAGCAGTGG |
| Mouse VPAC2 S | ACCAGCCAGCCTCCTGGGTT |
| Mouse VPAC2 AS | AGTGGACGCCAAACAGGGGGA |
| Human POLR2A S | GCAAGCGGATTCCATTTGG |
| Human POLR2A AS | TCTCAGGCCCGTAGTCATCCT |
| Human SGLT1 S | TAGATTTACCATGGCTGGACTCTTACT |
| Human SGLT1 AS | CACCTGGGCAAAATTTACAACTG |
| Mouse POLR2A S | GCCAAAGACTCCTTCACTCACTGT |
| Mouse POLR2A AS | TCCAAGCGGCAAAGAATGTC |
| Mouse SGLT1 S | CATTCCAGACGTGCACCTGTAC |
| Mouse SGLT1 AS | TCCAGGTCGATTCGCTCTTC |
| Mouse Villin S | GCCGCCATTACTGGTTTTAGTC |
| Mouse Villin AS | GTCTGAAGGAATGGGCAAATTACT |

**Table S3**: Activity of maltase and sucrase in WT mice fed LCHO or HCHO.

|  | Maltase^a^ | Sucrase^a^ |
| --- | --- | --- |
| LCHO | 2.40 ± 0.53 | 0.356 ± 0.070 |
| HCHO | 2.15 ± 0.22 | 0.368 ± 0.104 |

^a^ µmol/min/mg protein
